# Porous Open-Сell UHMWPE: Experimental Study of Structure and Mechanical Properties

**DOI:** 10.3390/ma12132195

**Published:** 2019-07-08

**Authors:** Alexei I. Salimon, Eugene S. Statnik, Michael Yu. Zadorozhnyy, Fedor S. Senatov, Dmitry D. Zherebtsov, Alexander A. Safonov, Alexander M. Korsunsky

**Affiliations:** 1 Center for Energy Science and Technology, Skoltech, Moscow 121205, Russia; 2 Institute of Physiologically Active Substances, RAS, Сhernogolovka 142432, Russia; 3 Center of Composite Materials, National University of Science and Technology “MISiS”, Moscow 119091, Russia; 4 Center for Design, Manufacturing and Materials, Skoltech, Moscow 121205, Russia; 5 Department of Engineering Science, University of Oxford, Parks Road, Oxford OX1 3PJ, UK

**Keywords:** porous UHMWPE, mechanical properties, DMA

## Abstract

Ultra-high molecular weight polyethylene (UHMWPE) is a bioinert polymer that is widely used as bulk material in reconstructive surgery for structural replacements of bone and cartilage. Porous UHMWPE can be used for trabecular bone tissue replacement, and it can be used in living cell studies as bioinert 3D substrate permeable to physiological fluids. It is important to develop techniques to govern the morphology of open-cell porous UHMWPE structures (pore size, shape, and connectivity), since this allows control over proliferation and differentiation in living cell populations. We report experimental results on the mechanical behavior of porous open-cell UHMWPE obtained through sacrificial removal (desalination) of hot-molded UHMWPE-NaCl powder mixtures with pore sizes in the range 75 µm to 500 µm. The structures were characterized using SEM and mechanically tested under static compression and dynamic mechanical analysis (DMA), bending, and tensile tests. Apparent elastic modulus and complex modulus were in the range of 1.2 to 2.5 MPa showing a weak dependence on cell size. Densification under compression caused the apparent elastic modulus to increase to 130 MPa.

## 1. Introduction

Ultra-high molecular weight polyethylene (UHMWPE) is a bioinert polymer that has been used in orthopedics as bearing material in artificial joints and in metal-on-UHMWPE articulation. Since the 1990s, owing to its combination of excellent bioinertness and mechanical performance, UHMWPE has attracted permanent interest as a suitable solution in the reconstructive surgery of cartilage in hip and knee joints [1] and in intervertebral discs [2], as well as in oral and maxillofacial surgery [3]. The current practice of implant fabrication involves hot molding and further subtractive mechanical shaping of bulk UHMWPE. High viscosity of molten UHMWPE precludes the use of 3D additive techniques to create complex structures similar to trabecular bone tissue, although it has been demonstrated that the sacrificial templating technique can introduce some additivity into manipulations with UHMWPE [4], at a precision and spatial resolution of about 500 µm. On the other hand, hot molding of UHMWPE-NaCl powder mixtures with subsequent desalination is especially suitable when feature sizes down to tens of µm are sought in complex structures. The flexibility is an attractive aspect of this technique, as the following types of structures can be produced using relatively simple equipment:

- Layered hybrids of porous and bulk UHMWPE;
- Porous components with the gradient of pore sizes;
- Components with controlled multimodal distribution of pore sizes;
- Structures containing loose or embedded particles or fibers of another material.

The ability to combine porous and bulk UHMWPE in a single tubular structure simulating the structure of natural radius and femur bones has been demonstrated [5], and osteoblast propagation into porous UHMWPE has been proven [6]. These advancements are important, since the strength and reliability of the bone-polymer interfacial transition zone can be improved through the creation of optimal structures with carefully adjusted mechanical properties.

The mechanical properties of oriented and non-oriented bulk UHMWPE have been extensively studied [7]. UHMWPE-based composites reinforced with auxetic TPU fibers, polyamides, montmorillonite clays, ceramic particles, and carbon nanotubes have been investigated using static and dynamic mechanical testing [8,9,10,11,12]. However, to the best of the authors’ knowledge, the static and dynamic mechanical properties of porous open-cell UHMWPE have not yet been reported, in contrast to close-cell low molecular weight PE foams which have been widely studied [13,14,15,16,17,18,19,20].

Permeable open cell structures created by the present technique can serve as the “guiding” substrate for living cells, governing the propagation and integration of cells into tissues, which has become possible on account of substrate structure engineering.

We report the results of SEM structure studies and static and dynamic mechanical testing at compression, bending, and tensile.

## 2. Materials and Methods

### 2.1. Sample Preparation

**Figure 1:**
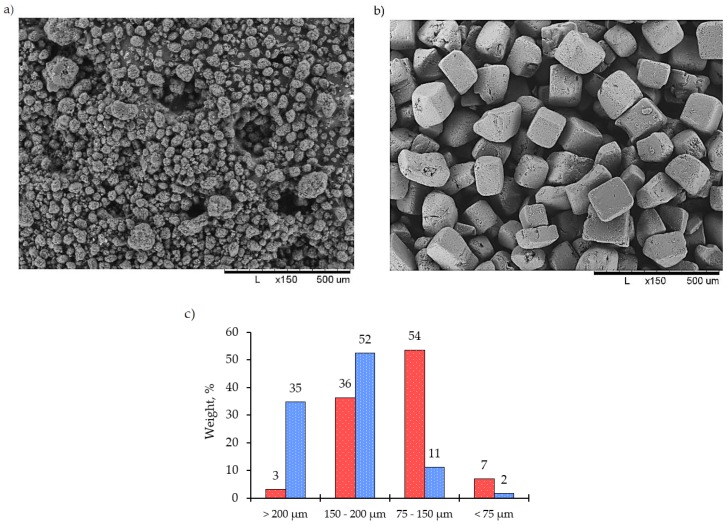
The appearance of the as-supplied powders of (**a**) ultra-high molecular weight polyethylene (UHMWPE); (**b**) rock salt; (**c**) The histogram of particle size distribution after sieving. Red bars—UHMWPE, blue bars—salt.

Pristine 4120 GUR UHMWPE powder (Ticona GmbH, Oberhausen, Germany) with an average molecular weight of 5·10^6^ g·mol^−1^ and food NaCl salt with a quasi-cubic shape and particle size ranging from 40 to 700 μm were classified with a set of sieves using a Fritsch Vibratory Sieve Shaker “Analysette 3 Pro” (Fritsch GmbH, Oberstein, Germany) as shown in Figure 1. To create a homogeneous distribution of pores with the chosen average cell size, powder mixtures with certain nominal particle sizes were prepared from the starting powders by sieving. The four fractions studied here had particle sizes in the ranges given below:

(a) Bigger than 200 μm;
(b) 150–200 μm;
(c) 75–150 μm;
(d) Smaller than 75 μm.

Mixtures of UHMWPE powder with loose salt and a weight ratio of 1:9, respectively, were gently stirred in a solid state in 500 ml corundum vials of Fritsch Planetary Ball Mill “Pulverisette 5” (Fritsch GmbH, Oberstein, Germany), using 8 mm diameter corundum balls. Portions of the mixtures were hot-molded at 50 MPa pressure and 180 °C temperature to obtain 3–5 cylinders with a diameter of 26 mm and length of 45 mm. The open cell porous structure with 80% of volume porosity was finally obtained through desalination in a vessel of Ultrasonic Cleaner “Elmasonic Denta Pro” (Elma Schmidbauer GmbH, Singen, Germany) using at least five bathes of distilled water at 60 °C for 48 hours. The completeness of the desalination was controlled gravimetrically and using SEM microscopy.

### 2.2. Compressive Testing

Compressive strength testing was performed using the universal Zwick/Roell Z010 machine (Zwick/Roell, Ulm, Germany), with a permanent traverse speed of 10 mm/min before 75% compression from an initial height.

Dynamic mechanical analysis was carried out in the air at a compression mode for cut cylindrical samples with diameter 6 mm and a range of heights between 6 and 8 mm. The analysis was performed at a heating rate of 5 K/min under harmonic loading, with 0.5% amplitude at 1 Hz using the DMA Q800 (TA Instruments, New Castle, DE, USA).

### 2.3. Tensile and Bending Testing

**Figure 2:**
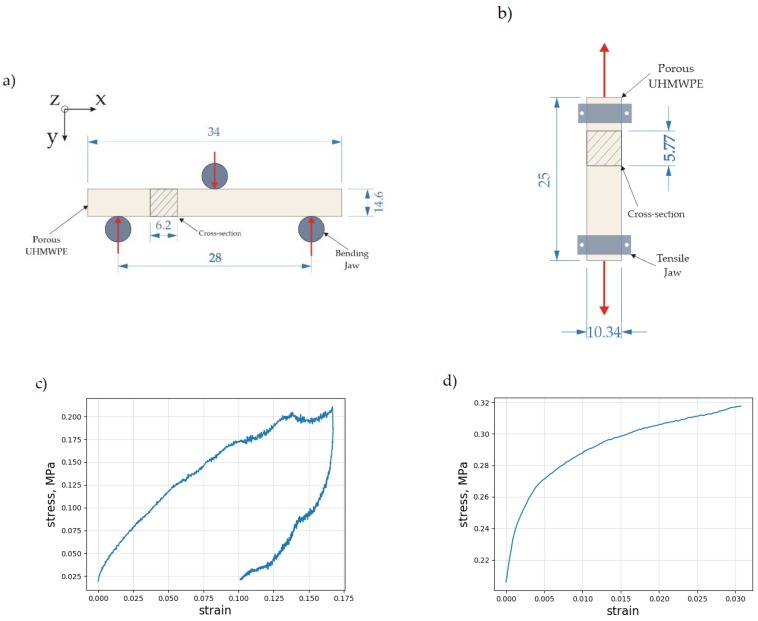
The schematic illustration of the three-point bending experiment (**a**) and tensile testing (**b**) Dimensions in millimeters. The stress–strain curves recorded during the three-point bending load application (**c**) and tensile testing (**d**), respectively.

The procedures for conducting three-point bending and tensile experiments were adopted from ISO standards 6892-1, 7438-2016, and ASTM standards E8 and E9. A set of three rectangular beams with a nominally identical porous UHMWPE structure were studied using the 1kN Deben Microtest device (Deben UK Ltd., London, UK) at a crosshead displacement speed of 1 mm/sec. Schematic illustrations of the tests are given in Figure 2a, b and c, d, respectively, along with the principal sample dimensions.

### 2.4. Scanning Electron Microscopy (SEM)

The preliminary structure studies were performed using an Altami MET 6C optical microscope (Altami, St. Petersburg, Russia). Pseudo-3D images were reconstructed from a stack of images using the Helicon Focus 7 software (version 7.5.5 for Windows 10).

The SEM investigation was conducted using scanning electron microscopes Hitachi TM-1000 (Hitachi, Tokyo, Japan) under the backscattered electron (BSE) regime.

## 3. Results and Discussion

**Figure 3:**
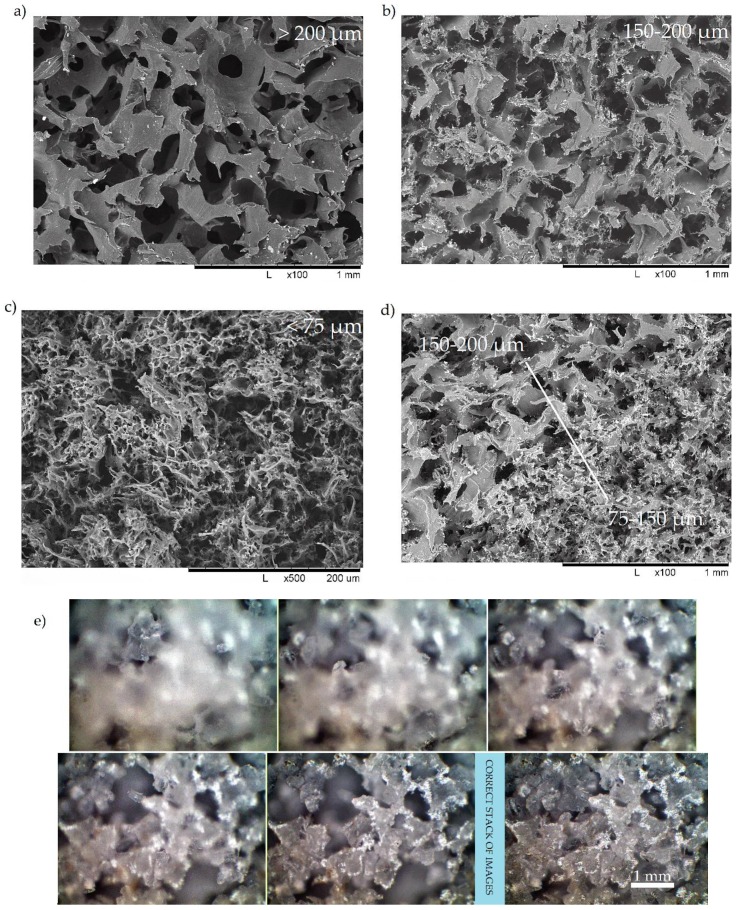
The scanning electron microscopy (SEM) images of porous UHMWPE uniform (**a**, **b**, **c**) and hybrid (**d**) structure fabricated from a powder having different average particle size, (**e**) a stack of optical images and deep focus pseudo-3D reconstructed image.

Figure 3a–c,e show a gradual diminishing of pore size and wall thickness when finer powders are used for the hot molding process, with no signs of coalescence or coarsening due to the low viscosity of UHMWPE. Moreover, Figure 3e was processed using deep focus or the “z-stacking” approach to creating images with an extreme depth of field, which could not be achieved by standard use of optical microscopes. The procedure of creating images with extreme depth of field consists of:

1. The “slices” (images with different focus distances) are acquired. Each image contains different parts of a specimen well focused.

2. Only the well-focused areas are used from each of the “slices” by the Deep Focus module. The resulting completely focused image is composed of these well-focused areas. Possible shifts and scale changes between the slices are automatically compensated.

An engineered hybrid structure with gradient variation of pore structure morphology, which is illustrated in Figure 3d, can be easily created in a single technological operation when layers of the UHMWPE-salt powder mixtures with controlled average particle size are stacked in the desired order.

**Figure 4:**
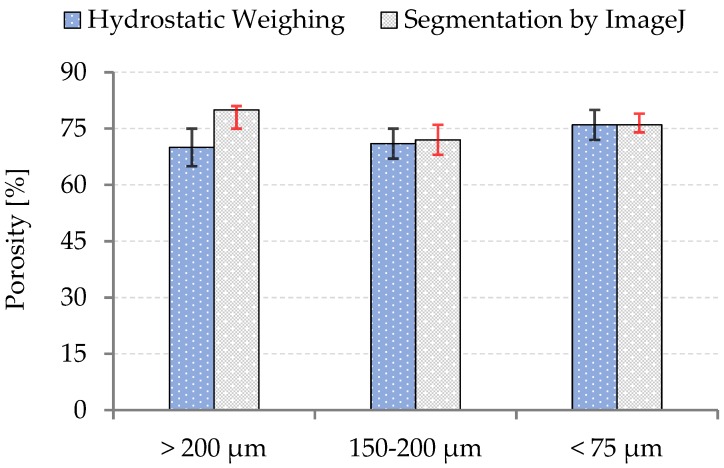
The porosity analysis of the prepared porous structures.

The porosity was calculated in accordance with the formula $P=\left(1-\rho_{por}/\rho_{bulk}\right)\cdot 100\%,$ where the densities of bulk and porous UHMWPE were determined through precise weighing and dimension measurements. The porosity was independently estimated using the analysis of SEM images. Segmentation was conducted with the help of open source Java image processing programs, such as Fiji (each piece of the pores was classified and essentially separated by a Gaussian blur filter (radius blurring is 2.0 pixel), and segmented using the Shanbhag threshold approach, respectively), as shown in Figure 4.

**Figure 5:**
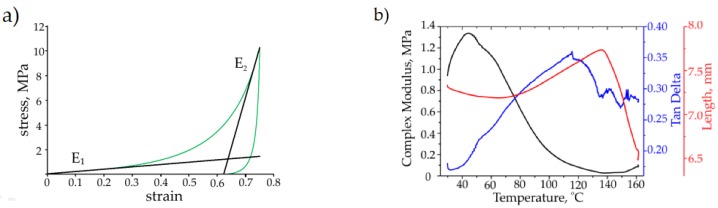
The static (**a**) and dynamic (**b**) mechanical properties of porous open-cell UHMWPE (fabricated from 75 to 150 µm powder fraction).

Figure 5a shows the studied stress-strain curve typical for open-cell porous UHMWPE at compression. The initial region corresponding to a range of 0 to 30% strain of apparent elastic behavior (here, modulus E_1_ is estimated using the slope of the tangent line) is related to the cascade of cell wall elastic collapse events [21]. At further compression, the sharp rise of stress is connected to porous structure densification and take-off of apparent stiffness (here, modulus E_2_ is calculated assuming no cross-section increment).

Figure 5b shows a typical evolution of complex modulus (E*) against temperatures in the range of 40 to 160 °C, as well as the recovery of the sample height and mechanical loss coefficient (tan δ). The decrease of the complex modulus occurred in the range of 40 to 120 °C, simultaneously with the sample height increase. The height of the sample rises more rapidly in the range of 80 to 145 °C (up to 147 °C—melting temperature), and then it promptly drops. We connected height enlargement to recovery phenomena, which are driven by the excess of elastic energy inherited during the sample cooling under mechanical loading after hot molding. Recovery is promoted by higher molecular chain mobility at elevated temperatures. The mechanical loss coefficient gradually grows in the range of 40 to 120 °C manifesting the enhanced contribution of inelastic intra- and inter-molecular motions.

Table 1 summarizes data on the apparent elastic moduli E_1_, E_2_, complex modulus, and tan δ at 40 and 100 °C for the compressive test and the Young’ modulus for tensile and bending experiments.

The value of Young’ modulus measured for porous UHMWPE (>200 mm fraction) under tension was equal to 1.35 ± 0.60 MPa, in good agreement with data from the compression tests. Bending tests yielded Young’ modulus values of 1.10 ± 0.54 MPa.

The estimation of the initial elastic modulus E_1_ could also be performed using the formula $E_{\mathrm{por}}\;=\;0.05\cdot E_{\mathrm{bulk}}\cdot {\left(\rho_{\mathrm{por}}/\rho_{\mathrm{bulk}}\right)}^{2}$ [22], for volume porosity of around 80%. For non-oriented bulk UHMWPE, the density was 0.95 g/cm^3^ and the Young’ modulus was about 1 GPa, leading to the estimated E_1_ modulus of 2.93 MPa for a material with straight cell walls of uniform thickness. The values of the experimentally measured elastic modulus E_1_ were distributed over a range that lay somewhat lower than the theoretical prediction, likely due to the variability in the structure and density of the porous structure as a consequence of fabrication. Specifically, the inhomogeneity of the starting distribution of particles within powders and the unevenness of molding pressure distribution may cause folding and cell wall thickness variation, resulting in the lower apparent rigidity. Further developments of the technique, such as more careful control over powder particle size and uniform mixing, will bring improvements in mechanical performance.

The significance of the findings reported in this paper consists of the demonstrated ability to vary the overall porosity level and pore size using different sacrificial rock salt powders. It was found that the overall mechanical properties, such as Young’s modulus, showed weak dependence on pore size, in agreement with the reports found in the literature [23]. It could be anticipated, however, that such parameters as the ultimate tensile strength (UTS) of porous UHMWPE may show stronger dependence on the pore size. This situation would be similar to the well-known observation in metals whereby grain refinement does not lead to a significant change in the elastic modulus, but it has a strong effect on the flow stress and/or UTS.

## 4. Conclusions

Desalination of hot molded UHMWPE-salt mixtures is a strong competitor for the conventional sintering of loose UHMWPE powder, providing stronger binding of powder particles and acceptable mechanical properties. Moreover, for gradient materials and hybrids, this technique opens a way for the intelligent tuning of properties through the layout of layers after controlling powder particle sizes. Elastic moduli in the range of 1 to 2.5 MPa in combination with biocompatibility are suitable for soft tissue engineering in reconstructive surgery and cell studies. We also contend that open-cell UHMWPE might be suitable in soft tissue engineering [24] and in sophisticated studies of living cell physiology when a bioinert and permeable 3D substrate is required [21]. On the other hand, another potential and modern application of sponge and bulk UHMWPE with collagen/hydroxyapatite (HAp) slurry is the development of multilayered scaffold suitable for cartilage-bone implant patches (bulk UHMWPE/open-cell porous UHMWPE/collagen with HAp layers is equal to cortical/trabecular/biocompatible layers, respectively).

## Figures and Tables

**Table 1:** The static and dynamic mechanical properties of porous open-cell UHMWPE.

Powder particle size, mm	Compression		Dynamic mechanical analysis			
	E1, MPa	E2, MPa	Complex Modulus E*, MPa		Tan δ	
			40 °C	100 °C	40 °C	100 °C
>200	1.80 ± 0.50	60 ± 25	4.70 ± 1.60	0.80 ± 0.22	0.185 ± 0.019	0.310 ± 0.012
150–200	1.96 ± 0.50	42.5 ± 22	6.84 ± 2.09	1.28 ± 0.43	0.207 ± 0.009	0.325 ± 0.002
75–150	1.26 ± 0.18	56 ± 7	1.13 ± 0.43	0.25 ± 0.10	0.173 ± 0.004	0.326 ± 0.009
<75	2.14 ± 0.45	64 ± 18	1.20 ± 0.62	0.36 ± 0.14	0.191 ± 0.017	0.314 ± 0.008
